# Supplementary material for: Fish Collagen Peptides Protect against Cisplatin-Induced Cytotoxicity and Oxidative Injury by Inhibiting MAPK Signaling Pathways in Mouse Thymic Epithelial Cells
Source: Mar Drugs. 2022 Mar 28;20(4):232. doi: 10.3390/md20040232 (PMC9032569; doi:10.3390/md20040232)
Supplement: Supplementary file 1 [file marinedrugs-20-00232-s001.zip › marinedrugs-1623944-supplementary.pdf]

Table S1. Western blot antibodies

Antibody	Source	Antibody type	Size (kDa)
Bcl-2	Abcam	Rabbit polyclonal	26
Bcl-xL	Abcam	Rabbit polyclonal	26
Bax	Abcam	Rabbit polyclonal	21
Bad	Abcam	Rabbit polyclonal	18
p38	Cell Signaling Technology	Rabbit monoclonal	40
p-p38	Cell Signaling Technology	Rabbit monoclonal	43
ERK	Cell Signaling Technology	Rabbit monoclonal	42,44
p-ERK	Cell Signaling Technology	Rabbit monoclonal	42,44
JNK	Cell Signaling Technology	Rabbit monoclonal	46,54
p-JNK	Cell Signaling Technology	Rabbit polyclonal	46,54
Cytochrome-c	Santa Cruze Biotechnology	Mouse monoclonal	15
Cyclin D1	Cell Signaling Technology	Rabbit monoclonal	36
CDK1	Abcam	Mouse monoclonal	34
β-actin	Santa Cruze Biotechnology	Mouse monoclonal	43
